# Round the Hospitals

**Published:** 1921-07-02

**Authors:** 


### ROUND THE HOSPITALS.

It will be seen from the results of the elections to the College Council that of the eight vacancies for England and Wales five are filled by re-elected members, and there are three new names, Viscountess Cowdray (who was at the head of the poll), Mr. C. E. Leo Lyle, M.P., and Miss E. G. Love. These are admirable additions to the Council's strength. Viscountess Cowdray's known deep interest in the College assured her election, and her proved business capacity in conjunction with the fact that she is not a member of the College cannot fail to bring to its counsels that outside inspiration which many of the College friends have for long desired. Mr. C. E. Leo Lyle, as a Member of Parliament and Chairman of Queen Mary's Hospital for the East-End, has already proved his qualifications to help the nursing profession, while Miss E. G. Love, as a private nurse, will represent interests that deserve more than the one private nurse representative already on the Council. Whilst congratulating the members on their choice, we are sure they will regret the retirement from the Council of Miss Cox Davies, who has done such yeoman service for the College since its foundation, and whose influence has been of incalculable importance. The volume of her work has also been great, for, despite her other numerous public duties, she attended no fewer than twenty-three out of a possible twenty-four Council meetings in one year; she, at any rate, cannot fail to rejoice in her freedom. The two vacancies for Scotland were filled by the re-election of Miss Gill and Professor Glaister, and those for Ireland by the re-election of Sir Andrew Beattie, and the election of Professor Johnston.

A correspondent complains that no matron or sister was called to give evidence before Viscount Cave's Committee of Inquiry into the finances of voluntary hospitals. She appears to be oblivious of the fact that no matron holds responsibility for the financial stability of her institution. Many and varied are the burdens which rest on the matron's devoted shoulders. Undoubtedly by her economy and vigilance she can do much to keep the wolf from the door. But from the gnawing anxiety about money which haunts the managers, as from the ceaseless struggle to enlarge the circle of subscribers which strains the secretary, she is free. She is seldom a skilled accountant, and her frequent failures when she takes on herself the burden of a private nursing home betray the fact that nothing in her previous career has prepared her for the business of budgeting for a large household of sick people. Into the management, economical or otherwise, of the hospitals, Viscount Cave's Committee was not commissioned to inquire.

Members and certificate-holders of the Incorporated Society of Trained Masseuses, and of the kindred Institute of Massage and Remedial Gymnastics, have shown themselves to the number of about 3,000 alive to the new privileges secured for them by amalgamation and by the attainment of their Charter. The Chartered Society of Massage and Medical Gymnastics has secured the allegiance of about half the joint membership enrolled before it came into being. With a view to enrolling on the first register, to be published in the autumn, all who are entitled to have their names appear in it, the committee have issued a circular to remind the laggards of the benefits they are missing by their failure to become registered members. It is a telling leaflet, and is well calculated to arrest attention. Probably some 30 to 40 per cent. of the members of the I.S.T.M. and the I.M.R.G. have discontinued work for one reason or another. But even deducting these, there must be several hundred masseurs and masseuses who inadvertently are giving opportunity the go-by. The receipt of this reminder should bring in a good proportion of them.

**ROUND THE HOSPITALS**—(*continued*).

Founder's Day at the Lord Mayor Treloar Cripples' Hospital at Alton was celebrated with more than ordinary good-will last Monday. The King and Queen sent a hearty message of sympathy in the work, and Queen Alexandra as Patroness of the Children's League also sent an inspiring message. Forty-five members of the City Corporation were present to support the life-work of their colleague, the founder. Among the interesting events must be mentioned the presentation of prizes to the senior and junior probationers by Mrs. Lloyd George, who also presented bonuses gained by some of the domestic staff for long service. The hospital should rank as an admirable preliminary and first-year training-school under the provisions of the General Nursing Council. The conditions are ideal for girls first entering on a nursing career, and the work is both varied and hopeful in its curative character.

---

A sum of approximately £3,000 has been raised by the ball at Lansdowne House organised by Marchioness Curzon of Kedleston in aid of Queen Victoria's Jubilee Institute for Nurses. In addition to lending the house Mr. Gordon Selfridge provided the marquee for the supper and the electric illumination of the house and grounds, which was on a very fine scale and contributed greatly to the pleasure of the evening.

---

A very attractive programme is promised for the two days' garden fête and sale of work on July 13 and 14 organised for the fourth year by the nursing staff of King's College Hospital in aid of their hospital. The latest ideas in side-shows will be exploited, and the stalls will include one devoted to dolls and others to vegetables and fruit, cakes and sweets. One boldly and proudly will label itself the "White Elephant" stall. There will be dancing each day from 5 to 9, a continuous variety entertainment, and exhibitions and demonstrations in the pathological and *x*-ray departments. The hospital will be open to inspection continuously.

---

This month should see a further step towards the provision of additional benefits by approved societies for their members. The Ministry of Health has already informed the societies that it will sanction schemes for bringing such benefits into operation, and is prepared to furnish model draft schemes to that end. Among the additional benefits suggested by the Ministry is that of the provision of nursing service for members of the societies, for which purpose a separate fund may be set aside. It will be remembered that so long ago as last February the question was raised by the Central Council for District Nursing in London, and in April this Council informed the approved societies which have members resident in London that the Council was in a position to arrange for adequate nursing for all such members. It therefore invited the societies to make contributions in aid of such work as they are enabled to do under Section 20 of the National Health Insurance Act or by way of additional benefits out of surplus funds. As the report of Lord Cave's Committee has pointed out, the complete freedom is recognised of the approved societies to grant financial aid to the voluntary hospitals in respect of insured persons, but the claim of the hospitals to a large measure of support remains a strong one. The same claim applies in the case of the services of district nursing associations, and we are glad to find that it has been recognised already in different parts of the country. Whether the recognition shall be by a contribution or donation rather than a scheme of insurance by payment in advance (with its contractual implication) or compulsory payment afterwards in respect of service rendered is merely a matter of arrangement.

---

Strong opposition to co-operation in the matter of nursing insured persons was the leading feature at the annual meeting of the Hanley Nursing Society on June 17, when Mr. Walton Stanley, President, was in the chair, supported by the Mayor of Stoke-on-Trent. The Committee have refused to join in the scheme proposed by Queen Victoria's Jubilee Institute for nursing the insured, and fall back on the original purpose of the Society "to give free sick nursing to the whole of the poor at Hanley." Both the chairman and Dr. Prendergast spoke against what was described as "a bureaucratic system," and the latter warned the subscribers that "if once they got into the claws of the National Health Insurance Act they would find their power swept away." It is much to be regretted that the matter should be regarded from this narrow standpoint.

---

A new stone cross of simple design and good proportions has replaced the temporary wooden one on Miss Edith Cavell's grave at Life's Green, in close proximity to Norwich Cathedral. It has been erected by Miss Cavell's sister, Mrs. Wainwright. The inscription runs: "To the pure and holy memory of Edith Cavell, who gave her life for England, October 12, 1915. Her name liveth for evermore."

---

Economy is the order of the day at most hospitals and institutions in these times, and those charged with the administration and management have been casting about for ways and means whereby expenditure may be brought into closer approximation with present and probable future income. At the Whitchurch Mental Hospital, Cardiff, the Medical Superintendent, Dr. E. Goodall, C.B.E., has just introduced various economies whereby a large saving is promised, and he has also indicated other directions in which savings may be made. One of the steps taken on the medical superintendent's recommendation is to be a general reduction of the staff's war bonuses from July 1. Married male nurses are to receive five shillings a week less, single male nurses six shillings a week less, female nurses four shillings a week less, and the domestic staff half-a-crown a week less.

